# 表兄妹HLA全相合异基因造血干细胞移植治疗急性髓系白血病1例

**DOI:** 10.3760/cma.j.cn121090-20240921-00361

**Published:** 2025-05

**Authors:** 启璐 徐, 焕文 马, 云 刘, 孟晨 刘, 丽娟 王, 学红 冉

**Affiliations:** 1 潍坊市人民医院血液内科，潍坊 261041 Department of Hematology, Weifang People's Hospital, Weifang 261041, China; 2 潍坊市人民医院急诊外科，潍坊 261041 Department of Emergency Surgery, Weifang People's Hospital, Weifang 261041, China

患者，女，24岁，因“齿龈肿胀伴全身散在出血点伴白细胞增高”于2019年4月于潍坊市人民医院诊断为急性髓系白血病-M5。骨髓染色体核型为46,XX[20]，MLL-AF6基因阳性，AML-MDS基因突变阴性。给予降白细胞、水化、碱化、保肝、抑酸、利尿、止血等治疗后加用DA方案（柔红霉素+阿糖胞苷）化疗1个周期，疾病未缓解，改以HA方案（高三尖杉酯碱+阿糖胞苷）化疗2个周期，中剂量阿糖胞苷、中剂量阿糖胞苷、IA（去甲氧柔红霉素+阿糖胞苷）方案化疗各1个周期，获得形态学完全缓解（CR），多参数流式细胞术检测微小残留病灶（MRD）未见异常髓系原始细胞。于2019年11月行异基因造血干细胞移植（供者为其表兄，HLA配型10/10全相合，O+供AB+，患者母亲与供者父亲、患者父亲与供者母亲为互为同胞兄妹）。采用改良BU-CY预处理方案（阿糖胞苷1.5 g·m^−2^·d^−1^，−10 d、−9 d；白消安0.8 mg/kg每6 h 1次，−8 d~−6 d；环磷酰胺1.8 g·m^−2^·d^−1^，−5 d、−4 d，司莫司汀250 mg/m^2^，−3 d）。环孢素A、霉酚酸酯联合短程甲氨蝶呤（MTX）预防急性移植物抗宿主病（GVHD）。前列腺素E1预防肝静脉闭塞症（VOD），复方磺胺甲噁唑预防卡氏肺囊虫病，更昔洛韦预防巨细胞病毒感染。输注单个核细胞11.2×10^8^/kg，CD34^+^细胞4.99×10^6^/kg。+11 d粒细胞植活，+15 d血小板植活。+15 d患者出现恶心、呕吐及双手皮疹、脱屑现象，考虑急性GVHD，加用甲强龙治疗好转。+25 d复查骨穿示CR，MRD阴性，MLL-AF6融合基因阴性，WT1 0％，嵌合状态分析报告为供者完全嵌合状态，染色体46,XY[10]。+34 d发生甲沟炎，给予抗感染并加用泼尼松0.5 mg·kg^−1^·d^−1^治疗好转，+41 d出院，+100 d停环孢素A。移植后4个月发生慢性GVHD（口腔、眼、肝脏）。予甲泼尼龙等治疗后好转。移植后随访3年余，多次复查血常规正常，骨穿示CR，MRD阴性，MLL-AF6基因阴性，骨髓染色体核型46,XY[20]，呈完全嵌合状态。

讨论：本例患者与其表兄HLA配型10/10全相合，供受者为三代表亲（三级亲属），虽不是同胞全相合，但祖父母一代有四位共同祖先。予以同胞全相合标准方案预处理，未使用抗胸腺细胞免疫球蛋白。移植过程顺利，未发生Ⅲ度以上GVHD，移植后原发病持续缓解3年余。

